# The impact of parental involvement on adolescent depression: a chain mediation effect based on emotional interaction and parent–child trust

**DOI:** 10.3389/fpsyg.2026.1656150

**Published:** 2026-04-22

**Authors:** Yanru Long, Yuchi Ma

**Affiliations:** Department of Sociology, School of Social Development, East China Normal University, Shanghai, China

**Keywords:** adolescent mental health, parental involvement, parental warmth, parent–child interaction, parent–child trust

## Abstract

**Introduction:**

Adolescent mental health problems have attracted growing attention in contemporary China. In a context where parents are often highly involved in their children’s education, it is particularly important to understand how parental involvement is associated with adolescent depression through everyday relational processes within the family. This study examined whether emotional interaction and parent–child trust mediate this association.

**Methods:**

Data were drawn from the 2022 wave of the China Family Panel Studies. After excluding cases with missing values, the final sample included 1,414 adolescents aged 10–15 years. Structural equation modeling and bootstrap mediation analysis were used to estimate the chain mediation effect of parental involvement on adolescent depression.

**Results:**

Parental involvement was significantly associated with lower adolescent depression, but this association was fully mediated, with no significant direct effect after the mediators were included. The chain mediation pathway of “Parental involvement → Positive emotional interaction → Parent–child trust → Adolescent depression” was significant. Negative emotional interaction did not mediate this association, but it was associated with lower parent–child trust and higher adolescent depression. In addition, parent–child trust showed a significant independent mediating effect between parental involvement and adolescent depression.

**Discussion:**

The findings suggest that the association between parental involvement and adolescent depression operates mainly through relational processes within the family. Positive emotional interaction and parent–child trust appear to be key mechanisms in this association. Family-based interventions should therefore pay greater attention to relationship quality, emotional communication, and trust within the parent–child relationship.

## 1 Introduction

Adolescence is a critical developmental stage during which young people experience significant physiological, cognitive, and social changes, making them particularly vulnerable to mental health problems. According to the World Health Organization, approximately 14% of individuals aged 10–19 worldwide have experienced anxiety, depression, and other mental health issues (World Health Organization, 2024). Our study focuses specifically on adolescents aged 10–15 years, based on the age range covered by the CFPS2022 sample used in our analysis. In China, adolescent depression is particularly prevalent (Quach et al., 2015), and intense academic competition places adolescents under substantial psychological pressure (Zhao et al., 2024). Given the close link between educational success and socioeconomic status (Du and Li, 2024), Chinese parents often hold high educational expectations for their children and are deeply involved in their education. In this context, parental involvement has become a salient family factor in adolescent development.

Parental involvement shows positive influence on adolescent depression. Previous research has shown that the association between parental involvement and adolescent depressive symptoms is influenced by multiple factors, including adolescents’ age, grade level, and parent-reported educational involvement (Liu et al., 2024). However, from the perspective of family ecological theory, the influence of parental involvement toward adolescents stems not only from the behavior itself, but also from how it is experienced within family processes (Bronfenbrenner, 1986). Similarly, family socialization theory posits that children’s perceptions are pivotal in determining the impact of parental influence on development (Grusec, 2011). In this regard, adolescents’ own perceptions often more accurately predict their psychological outcomes (Liu et al., 2022; Thomas et al., 2020). Thus, although parental involvement is a parental behavior, it may affect adolescent depression through their relational experiences within the family.

Among these relational processes, emotional interaction and parent–child trust appear to be especially important. Parental involvement can promote positive emotional interaction between parents and children (Holzer et al., 2024; Moè and Katz, 2018). Such positive emotional interaction may further cultivate parent–child trust, fostering a stable relationship and predicting lower levels of adolescent depression (Lo and Ma, 2025). These findings suggest that emotional interaction and parent–child trust are two key relational mechanisms underlying the association between parental involvement and adolescent depression. Therefore, our study constructs a chain mediation model to examine whether and how parental involvement influences adolescent depression through these two relational mechanisms. Theoretically, the study contributes to the literature by clarifying the relational processes linking parental involvement to adolescent mental health, thereby extending existing research beyond direct associations or academic behavioral explanations. Practically, the findings may inform family-based prevention and intervention efforts by underscoring the importance of fostering supportive emotional interaction and trust in the parent–child relationship.

## 2 Literature review and hypotheses

### 2.1 Parental involvement and adolescent depression

In this study, parental involvement is defined as specific parental behaviors within the family context aimed at supporting children’s educational development, including attention to academic progress, investment in educational resources, and daily interactions related to educational activities (Hill, 2022). Previous research has classified parental involvement into three categories: home-based involvement, school-based involvement, and academic socialization. Among these, home-based involvement is more consistent with the educational practices of Chinese parents (Hill and Taylor, 2004). Rather than participating extensively in school affairs, Chinese parents are more likely to support their children’s development at home through homework assistance, academic supervision, and emotional support (Xia, 2024). Accordingly, our study conceptualizes parental involvement as family educational behaviors embedded in the parent–child relational context.

Prior research has shown that parental involvement is associated with adolescents’ mental health (Liu et al., 2024; Wang and Sheikh-Khalil, 2014). Specifically, supportive parental involvement, including warm communication, emotional support, and fostering autonomy, has been associated with greater emotional well-being and fewer depressive symptoms (Chen et al., 2024; Kang et al., 2024; Wang et al., 2014). Furthermore, the psychological consequences of parental involvement are partly influenced by adolescents’ perceptions of it, and parental involvement is more likely to reduce depression when it is perceived by adolescents as supportive rather than controlling (Liu and Zhang, 2023). Therefore, the present study conceptualizes parental involvement primarily in terms of its supportive dimension and proposes the following hypothesis:

*Hypothesis* 1: Parental involvement is negatively associated with adolescent depression.

### 2.2 The mediating role of emotional interaction and parent–child trust

Parental involvement shapes patterns of emotional interaction between parents and adolescents. Higher parental involvement is associated with more positive emotional interaction, including open communication, expressions of warmth, and shared activities (Ji et al., 2022; Zong et al., 2018). By contrast, insufficient parental involvement may weaken supportive communication and emotional exchange, thereby increasing the likelihood of negative emotional interaction, such as tension, conflict, and emotional disengagement (Cohen et al., 2024).

Emotional interaction is an important family process with significant implications for adolescents’ mental health (Katsantonis and McLellan, 2024; Loncarevic et al., 2024). It includes both positive and negative forms of affective and communicative exchange between parents and adolescents (Peng, 2023). Positive emotional interaction can strengthen emotional bonds and protect adolescents from depression (Gao et al., 2024), whereas negative emotional interaction tends to undermine relationship quality and increase adolescents’ risk of depression (Hutchinson et al., 2019; Ouyang and Cheung, 2023). Thus, emotional interaction may mediate the association between parental involvement and adolescent depression in both positive and negative directions. Accordingly, this study proposes the following hypotheses:

*Hypothesis* 2: Positive emotional interaction mediates the association between parental involvement and adolescent depression, with a negative indirect effect.

*Hypothesis* 3: Negative emotional interaction mediates the association between parental involvement and adolescent depression, with a positive indirect effect.

Parental involvement plays an important role in shaping parent–child trust. Higher parental involvement reflects parents’ sustained attention, time, and support for their children’s development, helping adolescents perceive their parents as available, responsive, and trustworthy (Jiang et al., 2020). Through more frequent communication and closer daily engagement, adolescents are more likely to perceive parental understanding and support (Zhou et al., 2025). Moreover, parents with higher levels of involvement tend to be more aware of their children’s academic stress and performance and are better able to provide timely encouragement, which can further strengthen adolescents’ trust in their parents (Bempechat et al., 2022).

Parent–child trust is defined as adolescents’ perceived trust in and reliance on their parents’ words, promises, and behaviors (Zhang and To, 2024). Trust from parents can enhance adolescents’ sense of efficacy and serve as a protective factor against mental risks (Lambert et al., 2015; Ross et al., 2024). Higher levels of parent–child trust make adolescents more likely to express their emotions and seek support within the family, reducing the risk of adolescent depression (Rossi et al., 2019). Therefore, parent–child trust may mediate the association between parental involvement and adolescent depression. Accordingly, our study proposes the following hypothesis:

*Hypothesis* 4: parent-child trust mediates the association between parental involvement and adolescent depression, with a negative indirect effect.

**Figure 1:**
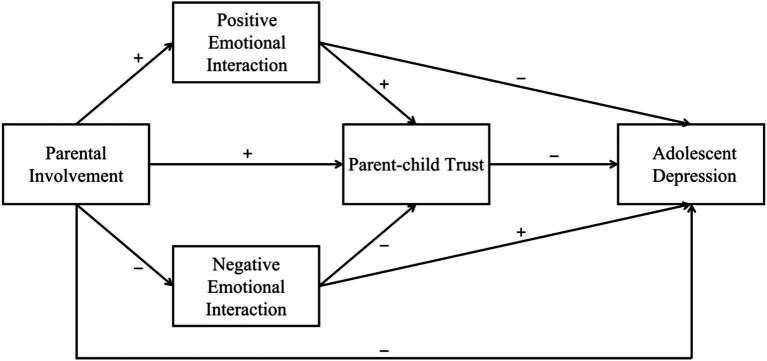
Conceptual framework of the chain mediation model.

Parental involvement may exert an influence on adolescent depression through a chained mediation process involving emotional interaction and parent–child trust. Emotional interaction reflects the direct relational exchanges through which parental involvement is expressed and experienced, whereas parent–child trust captures adolescents’ internalized evaluation of that relationship. Existing studies have shown that parental involvement shapes patterns of parent–child communication and emotional expression (Cheung and Pomerantz, 2012; Wang and Sheikh-Khalil, 2014), while adolescents’ perceived support, trust, and sense of belonging are closely related to mental health (Armsden and Greenberg, 1987; Chu et al., 2010). Positive emotional interaction can strengthen adolescents’ trust in their parents and lower depression risk, whereas negative emotional interaction may weaken such trust and increase depressive risk (Weisskirch, 2018). Thus, parental involvement may first shape the emotional tone of parent–child interaction, which then influences adolescents’ trust in their parents and ultimately affects adolescent depression. Because positive and negative emotional interaction function in opposite directions, they are expected to form two distinct chain pathways. Accordingly, this study constructs a chain mediation model of parental involvement, emotional interaction, parent–child trust, and adolescent depression (Figure 1) and proposes the following hypotheses:

*Hypothesis* 5: Positive emotional interaction and parent-child trust jointly mediate the association between parental involvement and adolescent depression, with a negative chain indirect effect.

*Hypothesis* 6: Negative emotional interaction and parent-child trust jointly mediate the association between parental involvement and adolescent depression, with a positive chain indirect effect.

## 3 Research methods

### 3.1 Data source and sampling

The data used in this study are derived from the China Family Panel Studies (CFPS), which is conducted by the Institute of Social Science Survey at Peking University. CFPS is a nationally representative social survey that comprehensively reflects dynamic changes in Chinese society across dimensions such as the economy, population, education, and health. In 2022, CFPS completed its seventh wave of nationwide tracking, covering all genetic members and their households since the baseline survey. CFPS2022 interviewed more than 23,000 households, with sample coverage spanning 25 provinces (municipalities and autonomous regions) across the country. The survey adopted a stratified, multi-stage probability proportional to size (PPS) sampling design, providing a nationally representative sample. After data cleaning and the removal of cases with missing values, a total of 1,414 adolescent individual observations were retained. The missing values mainly resulted from questionnaire items that were not applicable to some respondents or were left unanswered due to questionnaire routing, and no imputation procedure was applied.

### 3.2 Variable measurement

#### 3.2.1 Independent variable

The operationalization of parental involvement is based on actual circumstances reported in CFPS2022 regarding parents and their children during the previous or current academic term. It includes the following four items: (1) “When watching TV or video programs conflicts with your child’s study time, how often do you give up watching your preferred program to avoid affecting their learning?” (2) “How often do you discuss school-related matters with this child?” (3) “How often do you check this child’s homework?” (4) “How often does a family member take the initiative to contact the child’s teacher to learn about their situation?” Respondents were asked to evaluate each item using a 5-point Likert scale (1 = Never, 5 = Very Often). Confirmatory factor analysis was subsequently conducted and showed good model fit (CFI = 0.976, RMSEA = 0.065).

#### 3.2.2 Mediating variables

Emotional interaction is measured in two components: positive emotional interaction and negative emotional interaction, both based on adolescents’ self-reports. Positive emotional interaction is assessed with the item: “In the past month, how many times have you had heart-to-heart conversations with your parents?” Negative emotional interaction is measured by the item: “In the past month, how many times have your parents had quarrels?” Each item is coded into five frequency categories (0 = Never, 4 = 4 times or more). Parent–child trust is operationalized as adolescents’ perceived trust and support from their parents, represented by the item: “How much do you trust your parents? A score of 0 indicates no trust at all, and a score of 10 indicates complete trust.”

#### 3.2.3 Dependent variable

Adolescent depression is measured using a shortened version of the Center for Epidemiologic Studies Depression Scale (CES-D). Due to the number and complexity of items, CFPS2022 adopts an eight-item version to assess respondents’ actual experiences during the past week. The specific items include: “I felt depressed,” “I felt that everything I did was an effort,” “My sleep was poor,” “I felt happy,” “I felt lonely,” “I enjoyed life,” “I felt sad,” and “I felt that life could not go on.” Each item is rated according to frequency: rarely or none of the time (less than 1 day), some or a little of the time (1–2 days), occasionally or a moderate amount of the time (3–4 days), and most or all of the time (5–7 days), which are assigned scores from 1 to 4, respectively. The scale demonstrated acceptable internal consistency in the present sample (Cronbach’s *α* = 0.744). Confirmatory factor analysis indicates that the scale fits the model well (CFI = 0.989, RMSEA = 0.032).

#### 3.2.4 Control variables

Based on a review of relevant empirical studies, the following control variables are selected: adolescent gender (0 = female, 1 = male), adolescent age (10–15 years), household income (log-transformed), educational level (1 = illiterate/semi-illiterate, 6 = undergraduate and above), job (0 = agricultural work, 1 = non-agricultural work), party membership (0 = non-member, 1 = member), and marital status (0 = without spouse, 1 = with spouse). For the variables of educational attainment, occupation, and party membership, the highest response provided by either parent is used to represent the family’s actual situation. Since most variables were based on fixed-response categories with limited ranges, outlier problems were unlikely to be substantial. Household income was the main continuously reported variable and was log-transformed before analysis to address its skewed distribution. Detailed descriptive statistics are presented in Table 1 (with parental involvement and adolescent depression treated as latent variables; descriptive indicators are reported based on the mean scores of relevant questionnaire items).

**Table 1:** Descriptive statistics.

Variables name	Mean	Standard deviation	Minimum	Maximum
Adolescent depression	1.466	0.411	1.000	4.000
Parental involvement	3.169	0.718	1.000	5.000
Positive emotional interaction	1.841	1.649	0.000	4.000
Negative emotional interaction	0.792	1.246	0.000	4.000
Parent–child trust	9.306	1.349	0.000	10.000
Adolescent gender	0.524	0.500	0.000	1.000
Adolescent age	12.372	1.700	10.000	15.000
Household income	11.441	0.734	9.341	14.545
Educational level	3.403	1.205	1.000	6.000
Job	0.799	0.401	0.000	1.000
Party membership	0.127	0.333	0.000	1.000
Marital status	0.987	0.112	0.000	1.000
Sample size	1,414			

### 3.3 Analytical strategy

All data cleaning and statistical analyses were conducted using Stata 17.0, including bivariate correlation analysis, confirmatory factor analysis, structural equation modeling, and bootstrap mediation testing. This study first conducts bivariate correlation analysis to preliminarily explore the relationships among key variables. Structural equation modeling is then employed, specifying a total of six analytical paths:(1) Parental involvement → Adolescent depression;(2) Parental involvement → Positive emotional interaction → Adolescent depression;(3) Parental involvement → Negative emotional interaction → Adolescent depression;(4) Parental involvement → Parent–child trust → Adolescent depression;(5) Parental involvement → Positive emotional interaction → Parent–child trust → Adolescent depression;(6) Parental involvement → Negative emotional interaction → Parent–child trust → Adolescent depression. Finally, the mediating effects are tested using the bootstrap method, with 1,000 random samples drawn to assess whether the indirect effects fall within the 95% confidence interval.

## 4 Empirical results

### 4.1 Bivariate correlation analysis

We first present the bivariate correlations among the main variables (see the Table 2). Parental involvement was positively correlated with positive emotional interaction (*r* = 0.148, *p* < 0.05) and parent–child trust (*r* = 0.102, *p* < 0.05), suggesting that higher levels of parental involvement were generally accompanied by more supportive emotional exchanges and stronger trust in parents. Parent–child trust was positively correlated with positive emotional interaction (*r* = 0.116, *p* < 0.05) but negatively correlated with negative emotional interaction (*r* = −0.159, *p* < 0.05), indicating that a more positive emotional climate within the family was associated with stronger trust, whereas more negative interaction was associated with weaker trust. In addition, adolescent depression was negatively correlated with parental involvement (*r* = −0.077, *p* < 0.05) and parent–child trust (*r* = −0.197, *p* < 0.05), but positively correlated with negative emotional interaction (*r* = 0.193, *p* < 0.05). By contrast, the correlation between positive emotional interaction and adolescent depression was not significant (*r* = −0.014, *p* > 0.05). Thus, these bivariate results provide preliminary support for the proposed mediation framework, particularly the roles of parent–child trust and negative emotional interaction in adolescent depression.

**Table 2:** Bivariate correlation analysis results.

Variables	1	2	3	4	5
1. Adolescent depression	1.000				
2. Parental involvement	−0.077*	1.000			
3. Positive emotional interaction	−0.014	0.148*	1.000		
4. Negative emotional interaction	0.193*	0.012	0.109*	1.000	
5. Parent–child trust	−0.197*	0.102*	0.116*	−0.159*	1.000

*p < 0.05 (two-tailed test).

### 4.2 Chain mediation model test

**Figure 2:**
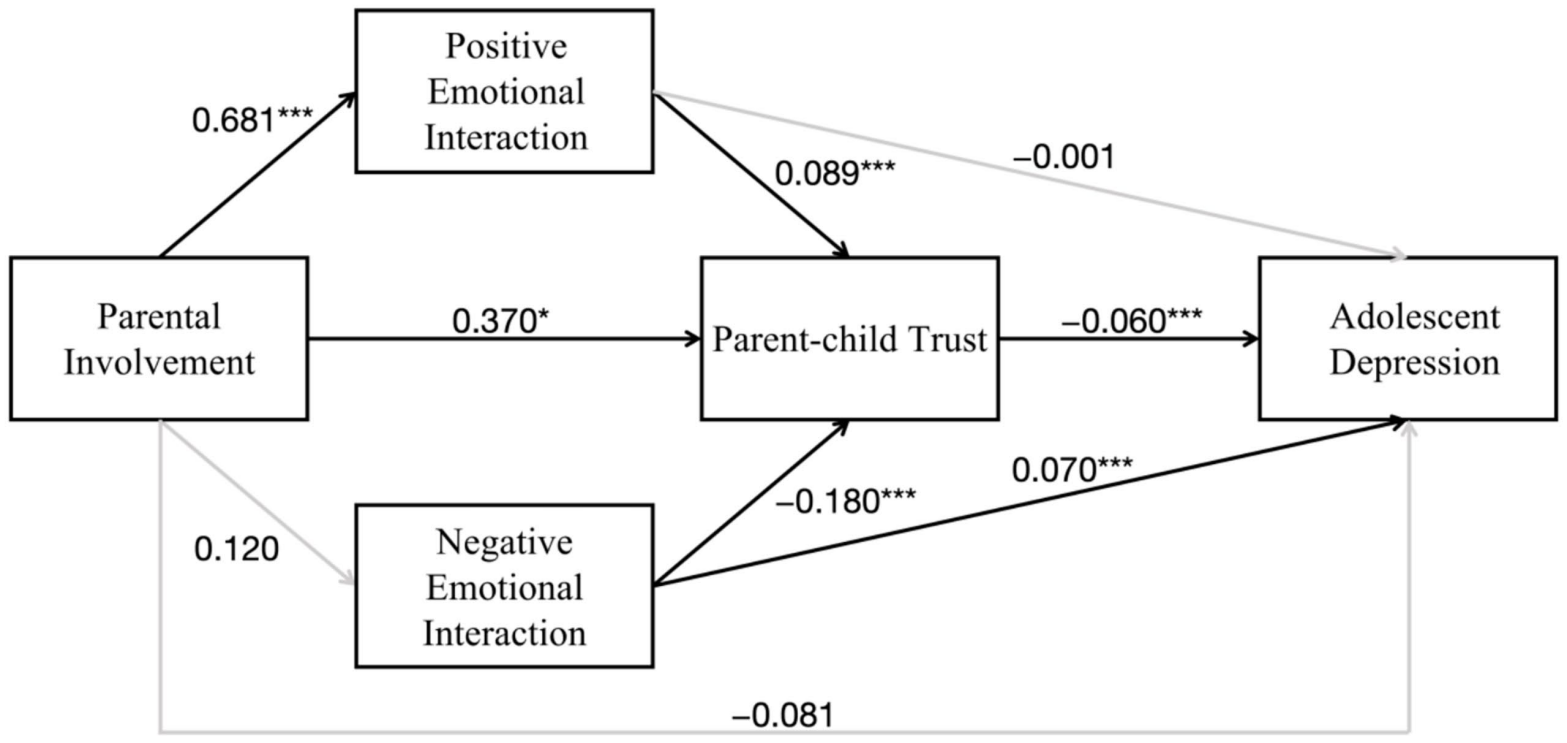
Estimated chain mediation model.

We further estimated a chain mediation model to examine how parental involvement is associated with adolescent depression (see the Figure 2). The results are presented in Table 3, with robust standard errors reported in parentheses. Model 1 is the baseline structural equation model without mediating variables, whereas Model 2 is the full chain mediation model. In Model 1, parental involvement was significantly negatively associated with adolescent depression (b = −0.101, *p* < 0.05), supporting Hypothesis 1. However, after the mediating variables were included in Model 2, the direct association between parental involvement and adolescent depression became non-significant (b = −0.081, *p* > 0.05), suggesting that the relationship may be fully mediated by the relational variables in the model.

**Table 3:** Results of the chain mediation model.

Variables	Model 1: Adolescent depression (without mediating variables)	Model 2: Adolescent depression
Parental involvement	−0.101* (0.049)	−0.081 (0.048)
Positive emotional interaction		−0.001 (0.008)
Negative emotional interaction		0.070*** (0.013)
Parent–child trust		−0.060*** (0.013)
Adolescent gender	−0.008 (0.026)	0.008 (0.025)
Adolescent age	0.007 (0.009)	−0.001 (0.009)
Household income	−0.027 (0.020)	−0.026 (0.019)
Educational level	−0.019 (0.013)	−0.027* (0.013)
Job	−0.003 (0.037)	−0.020 (0.037)
Party membership	−0.030 (0.034)	−0.011 (0.033)
Marital status	−0.091 (0.121)	−0.099 (0.124)
$\chi^{2}/\mathrm{df}$	2.138	2.340
RMSEA	0.028	0.031
CFI	0.956	0.940
TLI	0.945	0.917
SRMR	0.027	0.028

*p < 0.05, **p < 0.01, ***p < 0.001.

To test both the specific mediation effects and the chain mediation effects, all indirect paths between parental involvement and adolescent depression were further examined using the bootstrap method. The results are presented in Table 4. The total effect of parental involvement on adolescent depression was significantly negative (b = −0.097, 95% CI [−0.205, −0.014]), indicating that higher parental involvement was associated with lower levels of adolescent depression. After controlling for the mediators, the direct effect became non-significant (b = −0.081, 95% CI [−0.185, 0.004]), which is consistent with a full mediation pattern.

**Table 4:** Bootstrap analysis results.

Path	Effect size	Standard error	95% confidence interval	
			Lower	Upper
Total effect	−0.097	0.050	−0.205	−0.014
Direct effect	−0.081	0.048	−0.185	0.004
Parental involvement → Positive emotional interaction → Adolescent depression	−0.001	0.006	−0.013	0.011
Parental involvement → Negative emotional interaction → Adolescent depression	0.008	0.010	−0.010	0.027
Parental involvement → Parent–child trust → Adolescent depression	−0.022	0.011	−0.048	−0.004
Parental involvement → Positive emotional interaction → Parent–child trust → Adolescent depression	−0.004	0.002	−0.008	−0.001
Parental involvement → Negative emotional interaction → Parent–child trust → Adolescent depression	0.001	0.002	−0.002	0.005

Among the specific indirect effects, the pathway “Parental involvement → Parent–child trust → Adolescent depression” was statistically significant (b = −0.022, 95% CI [−0.048, −0.004]), supporting Hypothesis 4. This finding indicates that parental involvement may reduce adolescent depression by strengthening adolescents’ trust in their parents. In contrast, the indirect effects through positive emotional interaction alone (b = −0.001, 95% CI [−0.013, 0.011]) and through negative emotional interaction alone (b = 0.008, 95% CI [−0.010, 0.027]) were not significant; therefore, Hypotheses 2 and 3 were not supported. Regarding the chain mediation effects, the pathway “Parental involvement → Positive emotional interaction → Parent–child trust → Adolescent depression” was significant (b = −0.004, 95% CI [−0.008, −0.001]), supporting Hypothesis 5. However, the pathway “Parental involvement → Negative emotional interaction → Parent–child trust → Adolescent depression” was not significant (b = 0.001, 95% CI [−0.002, 0.005]), and thus Hypothesis 6 was not supported. Therefore, these findings show that parental involvement primarily affects adolescent depression through parent–child trust and through the chain pathway of positive emotional interaction and parent–child trust.

## 5 Discussion

Using CFPS2022 data, this study examines how parental involvement is associated with adolescent depression, with particular attention to the chain mediation roles of emotional interaction and parent–child trust. The main findings are as follows.

### 5.1 Parental involvement and adolescent depression in a fully mediated relationship

The first results indicate that the association between parental involvement and adolescent depression is fully mediated by emotional interaction and parent–child trust. In the baseline model without mediating variables, parental involvement was significantly negatively associated with adolescent depression, which supports Hypothesis 1. However, after emotional interaction and parent–child trust were introduced into the model, the direct effect of parental involvement became non-significant, while the total effect remained significantly negative, indicating a pattern of full mediation. This finding suggests that parental involvement may be associated with adolescent depression indirectly through relational processes within the family, particularly adolescents’ emotional experiences and trust in their parents. Within the sociocultural context of Chinese education, parental involvement often reflects a culturally valued form of familial responsibility centered on children’s educational development and future success (Park et al., 2011). A high level of parental involvement therefore signals substantial parental investment in time, effort, and patience. The finding of full mediation helps explain how these forms of parental investment may be linked to better mental health outcomes. One possible interpretation is that parental educational behaviors matter for adolescent depression insofar as they are reflected in adolescents’ everyday family interactions and internalized evaluations of the parent–child relationship. However, because this study does not compare parent-reported and adolescent-reported parental involvement, this interpretation should be treated with caution. Thus, the relationship between parental involvement and adolescent depression appears to involve not only behavioral factors, but also relational and perceptual processes.

### 5.2 The effects of emotional interaction on parent–child trust and adolescent depression

The second key conclusion concerns the role of emotional interaction in shaping parent–child trust and adolescent depression. Positive emotional interaction was positively associated with parent–child trust, whereas negative emotional interaction was negatively associated with parent–child trust and positively associated with adolescent depression. However, Hypothesis 2 was not supported, as positive emotional interaction did not directly mediate the association between parental involvement and adolescent depression. This suggests that positive emotional interaction alone is not sufficient to reduce depressive symptoms. Instead, its protective effect only emerges when such interaction is further internalized as a stable sense of trust between parent and child, which explains why Hypothesis 5 could be supported. By contrast, Hypothesis 3 was not supported because parental involvement was not significantly associated with negative emotional interaction, even though negative emotional interaction itself significantly predicted lower trust and higher depression. This indicates that negative emotional interaction is not a mechanism through which parental involvement affects depression, but a distinct relational risk factor. Short-term conflict or criticism can intensify emotional distress and increase the risk of depression in adolescents (Yang et al., 2025). Overall, positive emotional interaction appears to exert its protective role indirectly through trust, whereas negative emotional interaction functions more as a direct relational risk factor.

### 5.3 The effects parent–child trust as transmission mechanism

The third finding concerns the mediation pathways linking parental involvement to adolescent depression. Hypothesis 4 was supported, as the path “Parental Involvement → parent-child Trust → Adolescent Depression” showed a significant negative mediation effect. Hypothesis 5 was also supported, as the path “Parental Involvement → Positive Emotional Interaction → parent–child Trust → Adolescent Depression” showed a significant negative chain mediation effect. In contrast, Hypothesis 6 was not supported because the chain path involving negative emotional interaction and parent–child trust was not significant. These findings highlight the central role of parent–child trust in the relationship between parental involvement and adolescent depression, both as an independent mediator and as a key link through which positive emotional interaction exerts its protective influence.

The findings regarding the chain mediation pathway do not contradict previous studies; rather, they help clarify a possible mechanism linking parental involvement to adolescent depression. Specifically, the results suggest that the influence of parental involvement on adolescents’ mental health depends on the extent to which parental behavior is translated into adolescents’ psychological perceptions. This study further shows that adolescents are at greater risk of depression when they report lower levels of trust in their parents or experience frequent negative emotional expressions, such as quarrels. Therefore, the effect of parental involvement on adolescent depression depends not simply on the intensity of parental educational responsibility, but on how such involvement shapes parent–child interaction, emotional expression, and adolescents’ subjective experiences.

Accordingly, the present study suggests that understanding adolescent depression requires more than attention to the social environment and observable parental behavior. It also calls for an adolescent-centered perspective that emphasizes adolescents’ own responses to external influences. This perspective helps clarify the relational processes underlying adolescent mental health. In the context of Chinese family education, under the prevailing model of “high expectations and high investment” (Du and Li, 2024), family education should not be limited to academic supervision and encouragement. It should also involve the cultivation of healthy parent–child interaction patterns and the development of parent–child trust. In practice, this may include listening more carefully to adolescents’ emotional experiences, maintaining open communication during academic supervision, reducing criticism and harsh confrontation when conflicts arise, and providing reassurance and encouragement when adolescents experience stress. Such efforts may help parents move away from a predominantly authority-based style toward a more emotionally supportive and trust-based relationship, thereby offering more effective support for adolescent mental health.

### 5.4 Limitations

This study has several limitations. First, the data are cross-section, which limits our ability to draw causal inferences. Future research may incorporate panel data or experimental designs to further examine the causal relationship between parental involvement and adolescent depression. Second, the measures of emotional interaction and parent–child trust are based on adolescents’ self-reports. Future studies could incorporate parents’ reports for comparative analysis. Third, this study does not conduct heterogeneity analyses across geographic region, urban–rural location, or class stratification. From the perspective of family ecological theory, family processes are shaped by broader structural and contextual conditions (Bronfenbrenner, 1986). Therefore, the pathways from parental involvement to emotional interaction, trust, and depression may vary across different social environments. For example, differences in household resources, parenting environments, and educational competition may influence both how parental involvement is translated into supportive emotional interaction and parent–child trust and how these relational processes are associated with adolescent depression. Thus, the mediation paths identified in this study may not operate uniformly across different population groups, and future research should further examine such contextual heterogeneity.

## 6 Conclusion

This study highlights the roles of emotional interaction and parent–child trust in the association between parental involvement and adolescent depression. The findings indicate that parental involvement was associated with lower adolescent depression through two main mechanisms: an independent mediating effect of parent–child trust and a chain mediation pathway through positive emotional interaction and parent–child trust The results suggest that the potential benefits of parental involvement depend not only on whether parents participate in their children’s education, but also on whether such involvement is accompanied by supportive emotional interaction and a trusting parent–child relationship. In practical terms, efforts to support adolescent mental health may be more effective when they involve multiple stakeholders, including parents, teachers, and school counselors, and when they focus not only on increasing parental involvement itself but also on improving the quality of parent–child communication and trust. More broadly, the findings imply that family education guidance and school-based mental health support should place greater emphasis on relationship quality within the family as an important component of adolescent well-being.

## Data Availability

The raw data supporting the conclusions of this article will be made available by the authors, without undue reservation.
